# A new integrative model for the co-occurrence of non-suicidal self-injury behaviours and eating disorder symptoms

**DOI:** 10.1186/s40337-021-00508-3

**Published:** 2021-11-22

**Authors:** Isabel Krug, Mercedes Delgado Arroyo, Sarah Giles, An Binh Dang, Litza Kiropoulos, Tara De Paoli, Kim Buck, Janet Treasure, Matthew Fuller-Tyszkiewicz

**Affiliations:** 1 Melbourne School of Psychological Sciences, University of Melbourne Psychology Clinic, Redmond Barry, Level 7, North Melbourne, VIC 3051 Australia; 2 Department of Psychiatry, University Hospital Vall de’ Hebron, Barcelona, Spain; 3 Department of Psychological Medicine, Institute of Psychiatry and Neuroscience, Section of Eating Disorders, King’s College London, London, UK; 4 School of Psychology, Deakin University, Geelong, VIC Australia; 5 Centre for Social and Early Emotional Development, Deakin University, Burwood, VIC Australia

**Keywords:** Eating disorder, Disordered eating, Non-suicidal self-injury, Attachment, Schemas, Affect dysregulation, Impulsivity, Self-esteem, Body dissatisfaction

## Abstract

**Objective:**

The high co-occurrence of non-suicidal self-injury (NSSI) behaviours and eating disorder (ED) symptoms suggests these conditions share common aetiological processes. We assessed a new integrative model of shared factors for NSSI and ED symptoms, where affect dysregulation, impulsivity, self-esteem, and body dissatisfaction mediated the relationship between insecure attachment and maladaptive schemas and NSSI and ED symptoms. A further aim of the study was to assess whether the model behaved similarly across a clinical eating disorder (ED) and a community sample.

**Method:**

123 females with a lifetime ED diagnosis and 531 female individuals from the community completed an online survey, which included measures assessing the variables of interest. A cross-sectional single time point analysis was used.

**Results:**

Invariance testing indicated that the model was structurally non-invariant (different across groups). The proposed integrative model was a good fit for the ED group, but for the community sample only a revised model reached an acceptable fit. Both attachment and maladaptive schemas, included early in the model, were implicated in the pathways leading to ED and NSSI symptoms in the ED and community groups. In the community group, impulsivity, a mediator, was a shared predictor for NSSI and bulimic symptoms. No other mediating variables were shared by NSSI and ED symptoms in the two groups. Overall, the proposed model explained slightly more variance for the ED group relative to the community group in drive for thinness (*R*^*2*^ = .57 vs .51) and NSSI (*R*^*2*^ = .29 vs .24) but less variance in bulimic symptoms (*R*^*2*^ = .33 vs .39).

**Conclusion:**

We conclude that the current model provides only limited support for explaining the comorbidity between NSSI and ED symptoms. It is vital to consider both common (e.g., attachment and maladaptive schemas) and specific factors (e.g., impulsivity) to better understand the pathways that lead to the co-occurrence of NSSI and ED symptoms.

A new integrative model assessed whether emotion dysregulation, impulsivity, self-esteem, and body dissatisfaction were mediators in the relationship between insecure attachment and maladaptive beliefs about the world and the self and subsequent eating disorder and self-harm symptoms. A further aim was to assess whether the proposed model differed between a clinical eating disorder and a community sample. All participants were female and included 123 patients with a lifetime eating disorder and 531 individuals from the community. Participating individuals completed an online survey at one timepoint, which included measures assessing the variables of interest. The findings of the current study indicated that the proposed model was a good match for the clinical eating disorder sample, but for the community sample only a revised model yielded acceptable statistical fit. Both insecure attachment and maladaptive beliefs about the world and the self, included early in the model, were indirectly related to eating disorder and self-harm symptoms for both the eating disorder and the community groups. Impulsivity, a mediator, was the only shared predictor for self-harm, and bulimic symptoms in the community group. We conclude that the current model provides only limited support for explaining the comorbidity between self-harming behaviours and disordered eating symptoms.

**Supplementary Information:**

The online version contains supplementary material available at 10.1186/s40337-021-00508-3.

## Introduction

Non-suicidal self-injury (NSSI, e.g., cutting, burning, biting) is frequently observed among individuals with eating disorders (EDs), with a meta-analysis reporting the lifetime history of NSSI to be 27.3% amongst ED patients [1]. This high comorbidity suggests shared factors for both EDs and NSSI [2]. To explain the co-occurrence of these behaviours, Svirko and Hawton [3] and Claes and Muehlenkamp [2] proposed a conceptual model of risk, which includes adverse childhood events, affect dysregulation, impulsivity, low self-esteem, and dissociation. Although this model has received preliminary empirical support in the literature [4–6], results have demonstrated that the model only explains a small amount of variance in NSSI and ED symptoms [4]; suggesting the model might benefit from the inclusion of processes previously implicated in both EDs and NSSI. Considering the clinical implications of the comorbidity between NSSI and EDs, there is a need to identify shared factors and potential intermediary mechanisms underlying these behaviours so that effective prevention and early intervention efforts can be established. Two factors that are known to influence NSSI and EDs, yet to be tested in the context of a comprehensive, integrated model are insecure attachment and early maladaptive schemas [7, 8]. The current study is the first to extend the existing conceptual model of EDs and NSSI [2, 3], by including these variables.

### The co-occurrence between NSSI and ED behaviours

Evidence suggests that NSSI and ED behaviours are highly comorbid [1, 3]. In their meta-analysis of 29 studies, Cucchi et al. [1] found that 32.7% of people with bulimia nervosa (BN) and 27.3% of people with anorexia nervosa (AN) reported a lifetime history of NSSI behaviours. An earlier review found that 25.4 to 55.2% of ED patients reported at least one form of NSSI [3]. Although some studies found no differences in the prevalence of NSSI between ED subtypes [9, 10], most point to a higher prevalence of NSSI among ED subtypes characterised by binge-purging rather than restrictive behaviours [1, 11]. There is also evidence that NSSI is related to greater ED severity [12–14]. In non-clinical samples, research has also shown that individuals (especially adolescents) who engage in NSSI display significantly more eating pathology, including increased body dissatisfaction and binge/purge symptomatology than their non-NSSI peers [15, 16].

### Current models explaining the relationship between EDs and NSSI

Svirko and Hawton [3] and Claes and Muehlenkamp [2] provided a conceptual model of potential factors underlying the association between NSSI and EDs. In this proposed model, key distal risk factors such as major traumatic events, personality, culture, and a maladaptive family environment led to more proximal pathological processes, such as impulsivity, affect dysregulation, dissociation, self-critical cognitive styles (i.e., low self-esteem), need for control, and obsessive–compulsive tendencies. In turn, these factors are thought to lead to the development of NSSI and EDs [2, 3].

To date, only two studies have empirically evaluated this theoretical model in a clinical ED population [5, 6], and one study assessed the model in a university sample [4]. Muehlenkamp et al. [5] tested a simplified version of the model among 422 ED female inpatients. The model assessed whether childhood trauma, low self-esteem, psychopathology (anxiety and depression), dissociation, and body dissatisfaction predicted NSSI. Findings revealed that childhood trauma had an indirect relationship with NSSI, mediated by low self-esteem, psychopathology, body dissatisfaction, and dissociation. The study also found that dissociation and body dissatisfaction were crucial factors in accounting for individual differences in NSSI. Although the model was a good fit to the data, it accounted for only a modest amount of the variance in NSSI (15%).

In the second clinical study, Vieira et al. [6], assessed a range of risk factors (both precursors and pathological processes) in 245 female ED outpatients with and without NSSI. In line with the conceptual model [2, 3], experiences of physical and sexual abuse acted as distal risk factors for NSSI in EDs, which in turn, were mediated by more proximal risk factors. Specifically, the paths from physical abuse to NSSI and ED were mediated by negative self-evaluation, substance use, and suicide attempts [6].

The third empirical study assessed associations between emotional distress, emotion regulation, avoidance, NSSI, and ED psychopathology in 230 female undergraduates [4]. The study found that the relationship between emotional distress and avoidance was mediated by limited access to emotion regulation strategies. Again, although the model provided a good fit to the data, the percentage of variance accounted for was only 16% for NSSI and 26% for ED pathology. These studies provide preliminary support for the conceptual model proposed by Svirko and Hawton [3] and Claes and Muehlenkamp [2]. However, the modest variance in NSSI and ED accounted for by the proposed models suggest that other important factors related to NSSI, and ED may need to be incorporated to better explain the psychological processes which link NSSI and EDs.

While previous theories have focussed predominantly on intrapersonal factors related to NSSI and ED behaviours, considerable evidence suggests that both NSSI and EDs are associated with interpersonal, social, and cognitive difficulties which predispose and maintain these behaviours [17, 18]. Self-injury and ED symptoms frequently occur in interpersonal contexts and are the result of the cognitive interpretation of these relationships [1]. As such, incorporating interpersonal and cognitive factors may add explanatory power and enhance our understanding of the factors linking NSSI and EDs. Two such factors that have been broadly implicated in both NSSI and EDs but not yet tested as part of an integrated model are insecure attachment [7, 19] and early maladaptive schemas [11].

### Insecure attachment and early maladaptive schemas in EDs and NSSI

Attachment difficulties are considered salient features of EDs [18, 19] and NSSI [7, 20, 21]. Moreover, emotion dysregulation has been found to mediate the relationship between attachment difficulties and NSSI and EDs [20]. Despite the importance of insecure attachment to both EDs and NSSI, no study has yet assessed this potential interpersonal risk factor in the context of a broader model of NSSI and EDs.

One possible adverse consequence of attachment difficulties is the development of early maladaptive schemas [22], defined as implicit negative beliefs about oneself and one’s relationship with the environment that is self-perpetuating which are relatively stable over time [22, 23]. Research into schemas has predominantly drawn on Young’s model [22, 24], which suggests there are five schema domains that correspond to unmet emotional needs in childhood: (1) disconnection and rejection, (2) impaired autonomy and performance, (3) impaired limits, (4) other-directedness, and (5) over vigilance and inhibition.

ED patients typically endorse significantly more maladaptive schemas than healthy controls [25, 26]. Previous ED studies [11] have found significant associations between (1) cluster B personality disorders (narcissistic, antisocial, histrionic and borderline [27], and bulimic symptoms and the schemas of insufficient control, emotional deprivation and mistrust/abuse schemas; and between (2) cluster C personality disorders [avoidance, dependent and obsessive–compulsive, [27]], and restrictive ED symptoms and the schemas of failure to achieve, social undesirability, subjugation and unrelenting standards [28, 29]. These maladaptive schemas have also been associated with NSSI in both clinical ED patients [11] and non-clinical ED samples [30]. Therefore, the literature has identified insecure attachment and maladaptive schemas as essential precursors of both ED and NSSI. However, no known study has empirically investigated these constructs concurrently within an integrated model.

### The current study

**Fig. 1:**
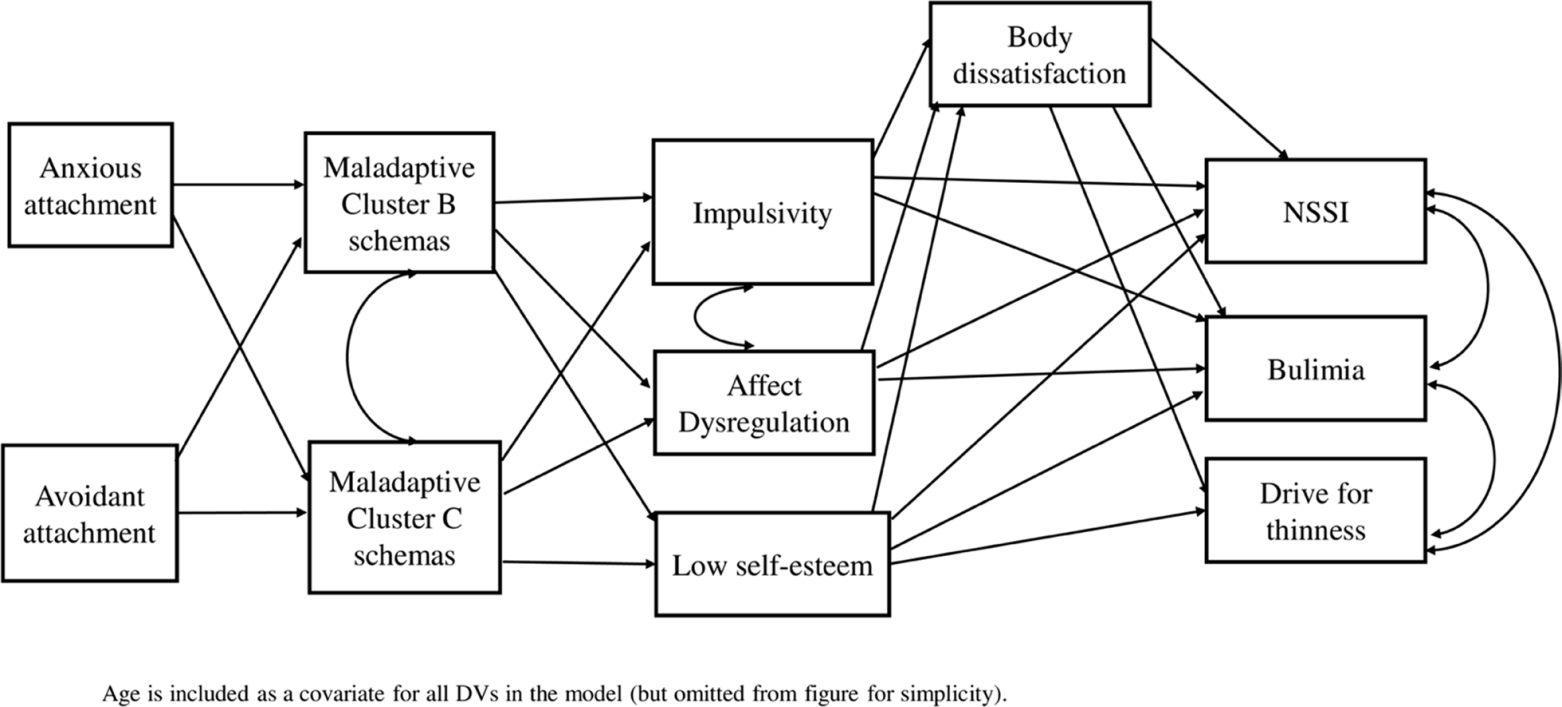
Proposed path model for NSSI and ED symptoms

The current study aimed to assess a novel extension of the conceptual model proposed by Svirko and Hawton [3] and Claes and Muehlenkamp [2] by integrating insecure attachment and the maladaptive schemas as distal risk factors for NSSI and EDs. Based on this model of NSSI and EDs (see Fig. 1), we expected that insecure attachment would be related to early maladaptive schemas, which in turn, would be associated with NSSI and ED behaviours through variables proposed in the existing conceptual model [2, 3], including affect dysregulation, impulsivity, self-esteem, and body dissatisfaction. Given that ED symptoms vary on a continuum independent of a clinical diagnosis [31], and individuals with NSSI exhibit significantly higher levels of eating pathology compared to non-NSSI individuals [15], this study also assessed whether the model was invariant across a clinical ED and a community sample.

In the proposed model, maladaptive schemas were summarized as schemas related to Cluster B (insufficient control, emotional deprivation, and mistrust/abuse schemas) and Cluster C (failure to achieve, social undesirability, subjugation, and unrelenting standards) personality disorders. These two schema subdomains represent underlying disturbances in the perception of interpersonal relationships. These schemas were chosen because previous studies (e.g., [11]) have found significant associations between these two clusters of schemas, and EDs, and to maximise model parsimony. Body dissatisfaction was placed as a mediating variable before the NSSI and ED variables, as body dissatisfaction has been recognised as one of the most predictive factors for disordered eating behaviours (e.g., [32, 33]).

Based on the reviewed literature we hypothesized that the clinical ED sample would present with more NSSI and ED symptoms and more significant paths in the model than the community sample. Furthermore, it was expected that the community would also display some NSSI and ED symptoms and significant paths, however these would be of significantly lower intensity in the community than in the ED model.

## Methods

### Participants

A total of 123 female participants with a lifetime ED diagnosis [54 Anorexia Nervosa (AN)-Restrictive, 18 AN-Binge Purge, 18 Bulimia Nervosa, 11 Binge Eating Disorder, 17 Other Specified Eating and Feeding Disorder, and 5 Unspecified Feeding or Eating Disorders] were recruited from two clinical ED units in Melbourne and other ED associations across Australia. Formal ED diagnosis was determined by psychiatrist report in clinical settings according to the Diagnostic and Statistical Manual of Mental Disorders (DSM-5) [27], and by self-report in other settings. The ED participants varied in treatment stage, with some being in residential care, while others attended outpatient treatment services (e.g., hospital or private psychologists) and yet others were not in treatment at the time of the assessment. Of the overall ED sample, 35% reported through a self-report questionnaire that they had recovered from their ED. The average age of onset of an ED diagnosis among this group was 15 years [Standard Deviation (SD) = 4.25].

A comparison group was recruited from the community and a first-year psychology university course. The community sample comprised 531 females from Australia. The mean age for the total sample was 22.48 years (*SD* = 8.13), and most of the participants were single, Caucasian, and currently studying at a University in Australia. The inclusion criteria for both samples included being female and at least 18 years of age. The decision to only include women in the study was three-fold. First, disordered eating attitudes and behaviours are commonly cited as higher in females (e.g., [27, 34]), hence there is greater urgency to address these issues for women. Second, there is a lack of research including males on the factors included in our integrative model of NSSI and ED symptoms, meaning that the validity of proposed pathways in the current model may be lacking for men. Third, the only previous studies [4–6] assessing similar models have also relied exclusively on female samples. The undergraduate students completed the survey for course credit whereas participants from the community and the clinical ED recruitment sites had the option to enter a draw to win an iPad mini. The data for this project was collected from 2014 to 2016.

### Measures

#### Sociodemographic

Information on participant age, height, weight, ethnicity, employment status, marital status, highest completed education, lifetime ED status, and age of ED onset were obtained through self-report. Body Mass Index (BMI) was calculated as the ratio of weight (kg) to height squared (m^2^).

#### Attachment style

Attachment style was measured using the Revised Experiences in Close Relationships scale [ECR-R; [35]]. The ECR-R is a 36-item self-report measure that assesses individual differences in *attachment anxiety* (18 items) and *attachment avoidance* (18 items). Items were scored on a 7-point scale from 1 (strongly disagree) to 7 (strongly agree). Items are scored on a 7-point scale from 1 (strongly disagree) to 7 (strongly agree). The ECR-R is a reliable measure of adult attachment [36].

#### Schemas

Early maladaptive schemas were assessed using seven of 15 subscales of the Young Schema Questionnaire – Short Form [YSQ-SF; [24]]: (1) insufficient self-control; (2) emotional deprivation; (3) mistrust/abuse; (4) failure to achieve; (5) social undesirability; (6) subjugation; and (7) unrelenting standards. All schema scales include 5 items, which are scored on a 6-point scale ranging from 1 (completely untrue of me) to 6 (describes me perfectly). A higher score is indicative of a more maladaptive belief. The seven subscales were collapsed into two schema Clusters according to previous research [11]: (1) maladaptive Cluster B schemas (emotional deprivation and mistrust/abuse) and (2) maladaptive Cluster C schemas (failure to achieve, social undesirability subjugation and unrelenting standards).

#### Affect dysregulation, self-esteem, impulsivity and NSSI symptoms

Affect dysregulation, self-esteem, impulsivity and NSSI symptoms were assessed using the Borderline Personality Questionnaire [BPQ; [37, 38]]. The BPQ is an 80-item self-report measure with items are scored as true or false. Four of the nine subscales were used in the current study: *impulsivity* (9 items), *affective instability* (to measure affect dysregulation, 10 items), *self-image* (to measure self-esteem, 9 items) and *suicide/self-mutilation* (to evaluate NSSI behaviours, 7 items). One item from the suicide/self-mutilation subscale was excluded (“I have made a suicide attempt in the past”) as it assessed suicide attempts and not self-injury behaviours, leaving this scale with a total of 6 items.

#### Eating disorder symptoms

Three subscales [*body dissatisfaction* (10 items), *bulimia* (8 items), and *drive for thinness* (7 items)] of the Eating Disorder Inventory 3 (EDI-3; [39]) were used to assess ED symptoms. Items are rated on a 6-point scale. The EDI-3 has demonstrated good psychometric properties [40] in previous studies.

### Procedure

Consenting adults were provided with a link to the online questionnaire hosted through Qualtrics Online Survey Software, which included the measures outlined above. Participants were asked to fill in the survey at their convenience. For the two participating clinics consenting, individuals were provided with this link during the intake interview. All data was collected in accordance with the Declaration of Helsinki and was approved by a University in Melbourne and two Hospitals in Melbourne. Participants were allowed to withdraw from the study at any time without affecting their treatment.

### Statistical analyses

Missing data analysis, descriptive statistics and univariate group difference analyses were conducted using IBM SPSS version 25. There was 1.25% missing data overall, and these missing values were imputed using expectation maximisation. We used t-tests and ANCOVAS (for adjusted analyses) for continuous variables, and chi-square tests for categorical variables to assess whether ED patients and the community sample differed on sociodemographic data or variables intended for the path analysis. Pearson correlations were undertaken to evaluate associations between variables before conducting the path analysis. Strength of the correlation was determined by Pearson’s *r*, with |.10|< *r* <|.30| indicating weak correlations, |.30|< *r* <|.50| medium correlations, and *r* >|.50| indicating a strong relationship [41].

The statistical framework applied to test the model was structural equation modelling. Path analysis using MPlus software was used to test the hypothesised model in Fig. 1. The model was run separately for the ED and the community sample to ascertain goodness of fit for each group. Following recommended criteria, non-significant chi-square (*p* > .05), Comparative Fit Index (CFI) values above .95, Root Mean Square Error of Approximation (RMSEA) below .10, and Standardised Root Mean Residual (SRMR) below .08 were used to indicate acceptable model fit [42]. In the event of poor model fit, modification indices were consulted, and recommended paths were added if theoretically or logically justifiable.

Given the marked differences in the initial model fit and proposed revisions for the ED and the community separately, a common model was not deemed suitable. Instead, final models are reported separately for each group. Model-implied mediation pathways were tested for significance using bias-corrected bootstrapping with 5000 bootstraps [43]. In the final models age was included as a covariate for all dependent variables. In the following results section only the significant paths are being outlined. Model parameters for non-significant paths can be provided on request from the corresponding author.

Assuming alpha is set at 0.05 (two-tailed) and power at 0.80, a sample size of 123 participants (reflecting the smaller of the two groups) was adequately powered to detect paths that uniquely account for at least 5% variance in any of the dependent variables, reflecting small effects. This sample also had power = 0.93 to detect poor overall model fit, defined as RMSEA = 0.10 relative to RMSEA = 0.01 [44].

## Results

### Sociodemographic characteristics

Significant group differences were observed for age, ethnicity, marital and employment status, and BMI, with the ED group being significantly more likely to be older, Caucasian, married, unemployed and having a lower average BMI than the community group. Conversely, the community sample contained significantly more Asian, single, and student participants than the ED group. There were no significant group differences on the highest level of education attained (Table 1).

**Table 1:** Sociodemographics of the sample

	Total (n = 654)	EDs (n = 123)	Community (n = 531)	Statistic^	*p*	Effect size*
Mean (SD)						
Age (years)	22.48 (8.13)	25.30 (7.59)	21.83 (8.11)	4.33	< .001	.44
BMI	21.76 (4.46)	20.92 (5.00)	21.95 (4.31)	2.11	.037	.22
N (%)						
Ethnicity						
Caucasian	301 (46.0)	99 (80.5)	202 (38.0)	87.73	< .001	.37
Aboriginal/Torres Strait	1 (0.2)	0 (0.0)	1 (0.2)			
Asian	249 (38.1)	7 (5.7)	242 (45.6)			
European	69 (10.6)	13 (10.6)	56 (10.5)			
Middle-Eastern	10 (1.5)	1 (0.8)	9 (1.7)			
African	5 (0.8)	0 (0.0)	5 (0.9)			
Hispanic	1 (0.2)	1 (0.8)	0 (0.0)			
Other	18 (2.8)	2 (1.6)	16 (3.0)			
Highest completed education						
Primary	2 (0.3)	1 (0.8)	1 (0.2)	7.73	.102	.11
Secondary	322 (49.2)	65 (52.8)	257 (48.4)			
Tertiary	264 (40.4)	39 (31.7)	225 (42.4)			
Postgraduate	66 (10.1)	18 (14.6)	48 (9.0)			
Marital status						
Single	432 (66.1)	73 (59.3)	359 (67.6)	24.74	< .001	.20
In a relationship	170 (26.0)	29 (23.6)	141 (26.6)			
Married	42 (6.0)	16 (13.0)	26 (4.9)			
Separated	5 (0.8)	4 (0.3)	1 (0.2)			
Divorced	3 (0.5)	1 (0.8)	2 (0.4)			
Widowed	2 (0.3)	0 (0.0)	2 (0.4)			
Employment						
Working full-time	71 (10.9)	21 (17.1)	50 (9.4)	50.50	< .001	.28
Working part-time	149 (22.8)	29 (23.6)	120 (22.6)			
Unemployed	43 (2.0)	23 (18.7)	20 (3.8)			
Student	391 (59.8)	50 (40.7)	341 (64.2)			

^t-test for continuous outcomes, chi-square for categorical outcomes

^*^Cohen’s d for comparison on continuous variables; Cramer’s V for comparison with categorical variables

### Group differences in study variables

Table 2 presents differences between the ED and community sample in the study variables included in the proposed integrative model of NSSI and ED. The adjusted values in Table 2 are evaluating group differences after controlling for the sociodemographic variables where differences had been found between the two groups. ED patients scored significantly higher than community individuals on all variables included in the integrative model and these differences remained significant after adjustment for the sociodemographic variables. In the ED sample, 54% presented with at least some NSSI symptoms, while this number was significantly lower in the community group with only 19.9% presenting with NSSI symptoms.

**Table 2:** Descriptive statistics and group difference tests unadjusted and adjusted for sociodemographics

	ED (n = 123)	Cronbach Alpha	Community (n = 531	Cronbach alpha	*Unadjusted*		*Adjusted	
					*t*	*p*	*t*	*p*
Mean (SD)								
Attachment anxiety	3.93 (1.13)	.93	3.51 (1.20)	.94	3.49	.001	4.76	< .001
Attachment avoidance	4.05 (1.25)	.95	3.30 (1.13)	.94	6.45	< .001	6.33	< .001
*Schema B Cluster*								
Insufficient self-control schema	16.09 (6.69)	.88	14.51 (5.84)	.88	2.41	.017	4.70	< .001
Emotional deprivation schema	14.00 (7.14)	.92	12.07 (6.32)	.90	2.96	.003	2.25	.025
Distrust /abuse schema	15.06 (6.96)	.91	12.83 (5.88)	.89	3.30	.001	4.54	< .001
*Schema C Cluster*								
Failure to achieve schema	19.08 (8.12)	.97	12.64 (6.62)	.95	8.18	< .001	10.32	< .001
Social undesirability schema	19.76 (7.32)	.94	12.86 (6.53)	.94	9.61	< .001	9.26	< .001
Subjugation schema	16.73 (7.15)	.91	11.68 (5.64)	.88	7.33	< .001	5.41	< .001
Unrelenting standards schema	23.05 (5.89)	.88	18.46 (5.77)	.85	7.92	< .001	7.22	< .001
Affect dysregulation	5.98 (2.92)	.84	4.08 (2.95)	.83	6.45	< .001	6.89	< .001
Low self-esteem	6.18 (2.90)	.87	3.49 (2.69)	.81	9.84	< .001	9.32	< .001
Impulsivity	2.50 (2.04)	.69	1.43 (1.53)	.60	5.44	< .001	5.06	< .001
NSSI	3.17 (2.03)	.84	1.19 (1.63)	.81	10.09	< .001	11.69	< .001
Drive for thinness	17.94 (8.54)	.91	9.38 (7.26)	.87	10.28	< .001	11.10	< .001
Body dissatisfaction	30.09 (12.28)	.93	17.68 (10.55)	.90	10.36	< .001	12.17	< .001
Bulimia	11.52 (9.45)	.91	5.85 (6.27)	.87	6.33	< .001	8.46	< .001

^*^Adjusted for age, BMI, ethnicity, marital and employment status, and education

Table 2 also presents the Cronbach values for all measures, which provided satisfactory (α = 0.60 for impulsivity for the community sample) to excellent values (α = 0.97 for failure to achieve schema for the ED sample). Given the relatively low Cronbach alpha value for the impulsivity subscale, we re-ran the model in a scenario that simulated what results would look like if the measure had better internal consistency. This information is included in Additional file 1. The results of these simulation analyses show that the results of our path-model do not change with improved Cronbach alpha values.

### Path analyses

Correlational analyses (see Table 3) were run separately for the ED and the community samples to examine bivariate relationships, which formed the basis for the path analyses. Significant correlations were found between all the variables included in the model for both the ED and the community groups, with predominantly moderate to large effect sizes.

**Table 3:** Correlation analyses of the variables of interest

	Attachment anxiety	Attachment avoidance	Affective dysregulation	Self-esteem	Impulsivity	NSSI	Body dissatisfaction	Bulimia	Drive for thinness	Maladaptive cluster B schemas	Maladaptive cluster C schemas
Attachment anxiety	–	.51*	.50*	.48*	.28*	.26*	.45*	.28*	.31*	.58*	.61*
Attachment avoidance	.36*	–	.45*	.47*	.28*	.18	.44*	.27*	.31*	.50*	.55*
Affective dysregulation	.46*	.21*	–	.59*	.28*	.44*	.59*	.30*	.48*	.60*	.61*
Self-esteem	.53*	.34*	.56*	–	.24*	.26*	.64*	.29*	.64*	.50*	.74*
Impulsivity	.23*	.22*	.35*	.25*	–	.29*	.41*	.47*	.27*	.44*	.30*
NSSI	.29*	.22*	.41*	.38*	.30*	–	.35*	.26*	.36*	.42*	.32*
Body dissatisfaction	.36*	.27*	.36*	.49*	.30*	.26*	–	.46*	.73*	.49*	.58*
Bulimia	.29*	.24*	.36*	.41*	.41*	.27*	.56*	–	.43*	.38*	.34*
Drive for thinness	.29*	.15*	.33*	.38*	.27*	.26*	.71*	.61*	–	.40*	.54*
Maladaptive cluster B schemas	.51*	.39*	.55*	.61*	.39*	.41*	.40*	.43*	.35*	–	.68*
Maladaptive cluster C schemas	.51*	.32*	.54*	.70*	.28*	.41*	.44*	.40*	.37*	.79*	–

^*^Correlation is significant at the 0.01 level (2-tailed). Correlations for the ED sample appear above the diagonal, and correlations for the community sample appear below the diagonal

#### ED group

**Fig. 2:**
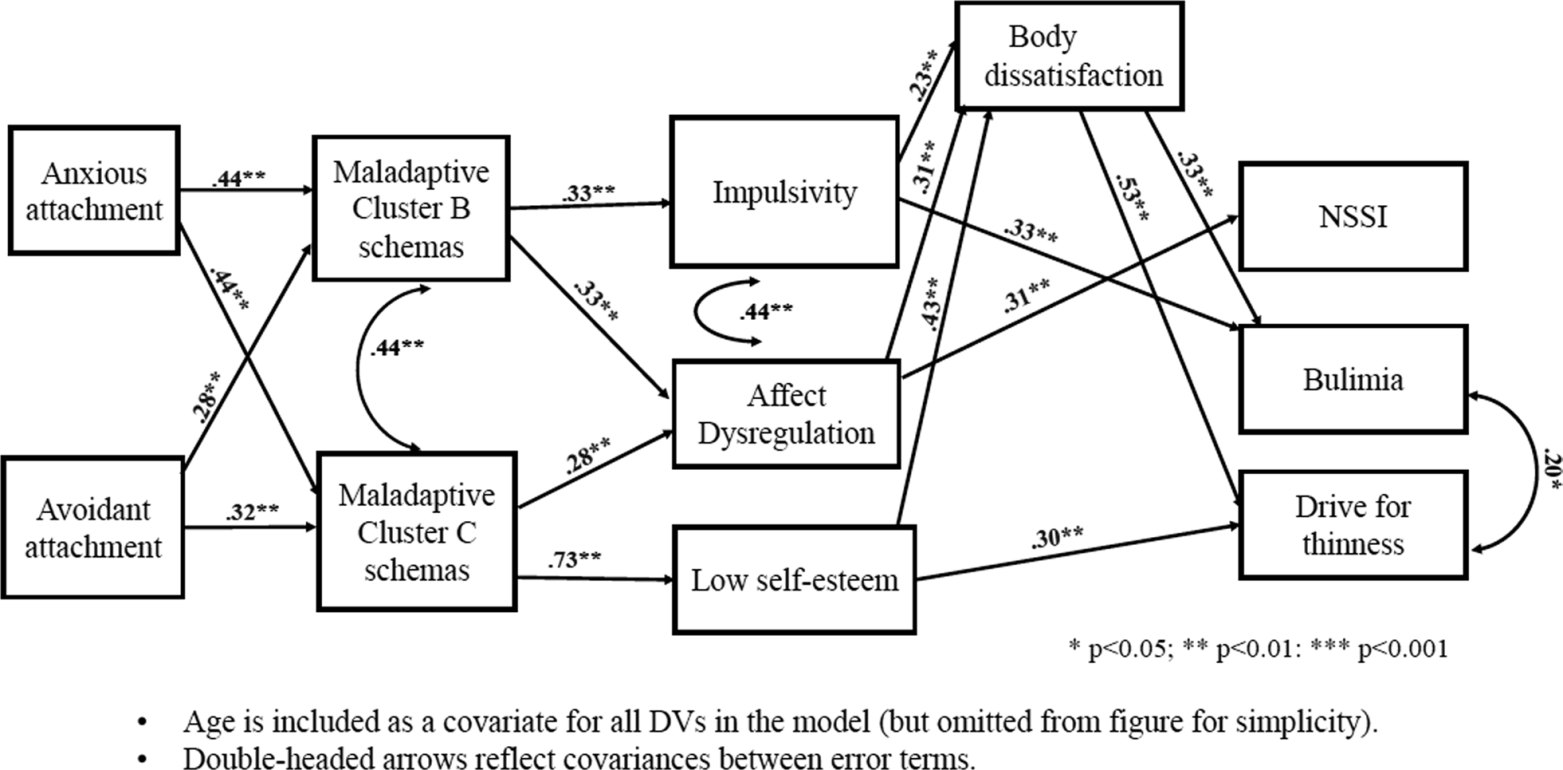
Proposed model results for the ED group

The proposed model had good overall fit; chi square _(df=26)_ = 25.22, *p* = 0.51, CFI = 1.00, RMSEA = 0.000, SRMR = 0.032. In terms of the key outcome variables in the model (right-most variables in Fig. 2), the predictor variables accounted for over one-quarter of the variance in NSSI, with significant unique contributions from affect dysregulation and age. Bulimic symptoms were uniquely predicted by body dissatisfaction, impulsivity, and age. In total, the predictors accounted for 33% of the variance in bulimic symptoms. The model accounted for 57% of the variance in drive for thinness, with significant unique contributions from self-esteem and body dissatisfaction. Regarding the associations among these outcome variables, the relationships between NSSI and bulimia and drive for thinness were non-significant after controlling for the predictors in the model. Bulimia and drive for thinness were significantly related.

In terms of the variables that functioned as mediators in the model (progressing from right to left in Fig. 2), the predictors combined accounted for 52% of the variance in body dissatisfaction, with significant contributions from impulsivity, affect dysregulation, and self-esteem. Impulsivity was significantly predicted by maladaptive Cluster B schemas. In total, 20% of the variance in impulsivity was accounted for in this model. The model accounted for 56% of the variance in self-esteem; with maladaptive Cluster C schemas being the only significant predictor.

Affect dysregulation was significantly predicted by age, maladaptive Cluster B schemas and Cluster C schemas, with 47% of the variance in affect dysregulation accounted for overall. Maladaptive Cluster B and C schemas had 39% and 45%, respectively, of their variance explained. Both maladaptive schema Clusters B and C were predicted by anxious attachment, and avoidant attachment. Maladaptive Cluster B and Cluster C schemas were significantly, positively related.

#### Community group

**Fig. 3:**
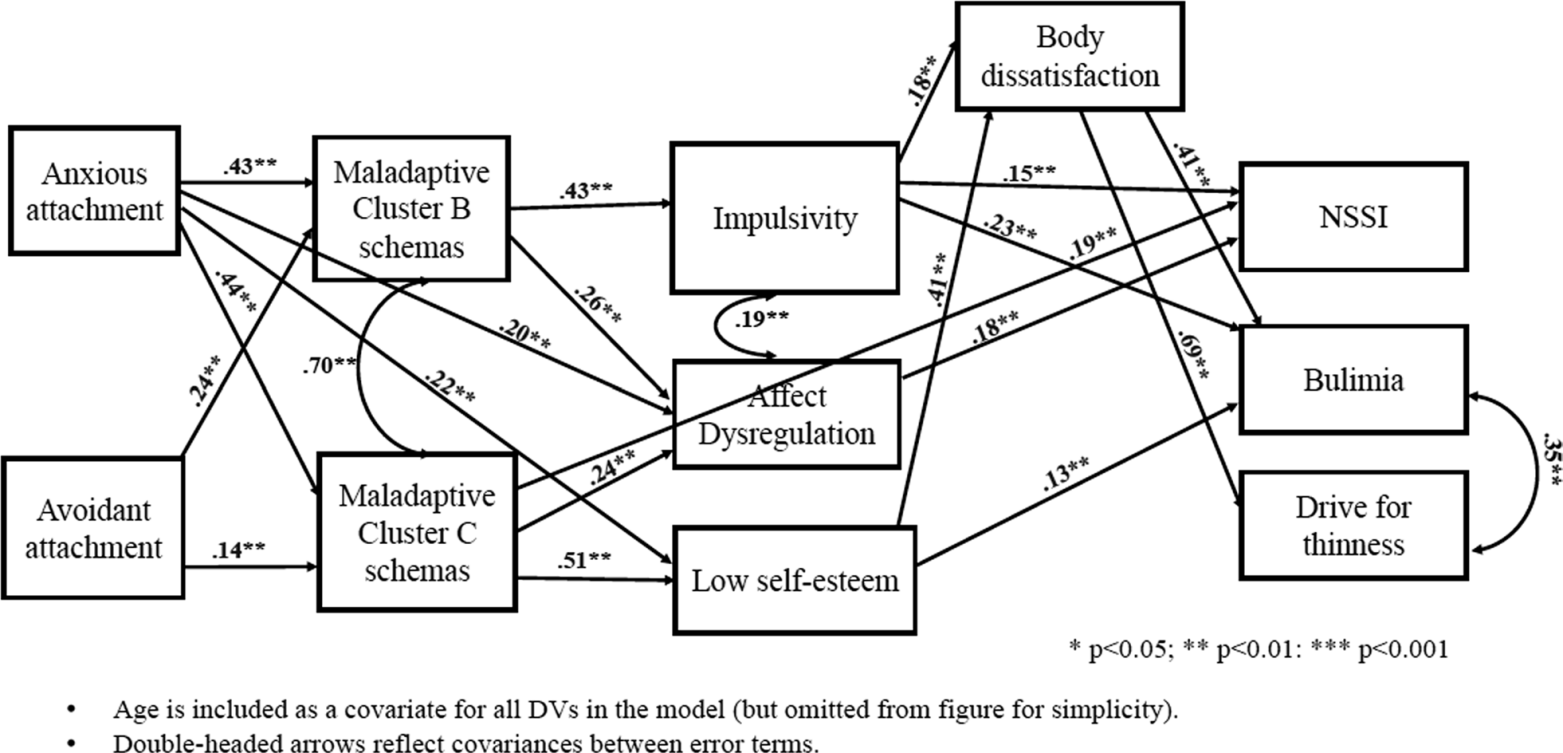
Proposed model results for the community group

The proposed model was a poor fit overall for the community group; chi square_(df=26)_ = 144.98, *p* < 0.001, CFI = 0.949, RMSEA = 0.093, SRMR = 0.054. Inspection of the modification indices identified four plausible paths to add to the model to improve fit: (1) a covariance term between affect dysregulation and self-esteem, (2) anxious attachment predicting affect dysregulation, (3) anxious attachment predicting self-esteem, and (4) maladaptive Cluster C schemas directly predicting NSSI. With these revisions, the model had acceptable fit; chi square_(df=22)_ = 51.82, *p* < 0.001, CFI = 0.987, RMSEA = 0.051, SRMR = 0.033. Relationships among modelled variables are reported below for this refined model working from the main outcome variables (right-most in Fig. 3) to mediators (right to left in Fig. 3).

Almost one-quarter (24%) of the variance in NSSI was accounted for, with significant unique contributions from affect dysregulation, impulsivity, and maladaptive Cluster C schemas. Bulimic symptoms were uniquely predicted by body dissatisfaction, impulsivity, and self-esteem. In total, the predictors accounted for 39% of the variance in bulimic symptoms. Over half the variance (51%) in drive for thinness was accounted for by the model, with body dissatisfaction demonstrated a significant unique contribution. The relationships between NSSI and bulimia and drive for thinness were non-significant after controlling for predictors in the model. Bulimia and drive for thinness remained significantly related.

Twenty-seven percent of the variance in body dissatisfaction was accounted for by its predictors combined, with significant contributions from impulsivity and self-esteem. Impulsivity was significantly predicted by maladaptive Cluster B schemas and age. In total, 16% of the variance in impulsivity was accounted for in this model. The model accounted for 53% of the variance in self-esteem; maladaptive Cluster C schemas and anxious attachment were the only significant predictors.

Affect dysregulation was significantly predicted by maladaptive Cluster B and Cluster C schemas, and anxious attachment, with and 36% of the variance in affect dysregulation accounted for. Maladaptive Cluster B and Cluster C schemas had 31% and 29% of their variance explained, respectively. Anxious and avoidant attachment were significant predictors of maladaptive Cluster B schemas and maladaptive Cluster C schemas. Age was also a significant predictor of maladaptive Cluster C schemas. Maladaptive Cluster B and Cluster C schemas were significantly and positively related, as was impulsivity with affect dysregulation.

### Indirect effects

#### ED group

The total indirect effect from the attachment variables to ED outcomes showed that anxious attachment on bulimia (*ß* = 0.14, 99% CIs: 0.05, 0.26), drive for thinness (*ß* = 0.24, 99% CIs: 0.12, 0.39), and NSSI (*ß* = 0.13, 99% CIs: 0.03, 0.26) were significant for the ED group. As shown in Table 4, the relationship between a.) anxious attachment and bulimia and b.) anxious attachment and drive for thinness was mediated by Cluster B and C, self-esteem, impulsivity, affect dysregulation, and body dissatisfaction. The relationship between anxious attachment and NSSI was mediated by Clusters B and C, affect dysregulation and self-esteem.

**Table 4:** Standardised coefficients from analyses testing indirect effect pathways from insecure attachment to NSSI and ED symptoms for the ED and the community groups

*Eating Disorder Group*		
Pathway	*ß*	99% CIs
Anxious → cluster B → impulse → bulimia	.06	.02, .14
Anxious → cluster B → instability → body dissatisfaction → bulimia	.01	.00, .05
Anxious → cluster C → instability → body dissatisfaction → bulimia	.02	.00, .05
Anxious → cluster C → self-esteem → body dissatisfaction → bulimia	.05	.01, .12
Anxious → cluster B → impulse → body dissatisfaction → bulimia	.02	.00, .05
Avoidant → cluster B → self-esteem → bulimia	.04	.01, .11
Avoidant → cluster B → instability → body dissatisfaction → bulimia	.01	.00, .04
Avoidant → cluster C → instability → body dissatisfaction → bulimia	.01	.00, .04
Avoidant → cluster C → self-esteem → body dissatisfaction → bulimia	.03	.01, .10
Avoidant → cluster B → impulse → body dissatisfaction → bulimia	.01	.00, .03
Anxious → cluster C → self-esteem → drive for thinness	.10	.02, .22
Anxious → cluster B → instability → body dissatisfaction → drive for thinness	.02	.01, .07
Anxious → cluster C → instability → body dissatisfaction → drive for thinness	.03	.01, .07
Anxious → cluster C → self-esteem → body dissatisfaction → drive for thinness	.07	.03, .16
Anxious → cluster B → impulsivity → body dissatisfaction → drive for thinness	.02	.01, .07
Avoidant → cluster C → self-esteem → drive for thinness	.07	.01, .17
Avoidant → cluster B → instability → body dissatisfaction → drive for thinness	.02	.00, .05
Avoidant → cluster C → instability → body dissatisfaction → drive for thinness	.02	.00, .05
Avoidant → cluster C → self-esteem → body dissatisfaction → drive for thinness	.05	.01, .13
Avoidant → cluster B → impulsivity → body dissatisfaction → drive for thinness	.02	.00, .05
Anxious → cluster B → instability → NSSI	.04	.01, .14
Anxious → cluster C → instability → NSSI	.05	.01, .14
Avoidant → cluster B → instability → NSSI	.03	.00, .09
Avoidant → cluster C → instability → NSSI	.04	.01, .10
*Community Group*		
Anxious → self-esteem → bulimia	.03	.01, .06
Anxious → cluster C → self-esteem → bulimia	.03	.01, .06
Anxious → cluster B → impulse → bulimia	.04	.02, .07
Anxious → self-esteem → body dissatisfaction → bulimia	.04	.02, .06
Anxious → cluster C → self-esteem → body dissatisfaction → bulimia	.04	.02, .06
Anxious → self-esteem → body dissatisfaction → drive for thinness	.06	.03, .10
Anxious → Cluster C → self-esteem → body dissatisfaction → drive for thinness	.06	.04, .10
Anxious → cluster B → impulse → body dissatisfaction → drive for thinness	.02	.01, .04
Anxious → instability → NSSI	.04	.01, .08
Anxious → cluster C → NSSI	.08	.01, .16
Anxious → Cluster B → instability → NSSI	.02	.01, .05
Anxious → Cluster C → instability → NSSI	.02	.01, .05
Anxious → Cluster B → self-esteem → NSSI	.03	.01, .06
Avoidant → cluster C → self-esteem → bulimia	.01	.00, .03
Avoidant → cluster B → impulse → bulimia	.02	.01, .05
Avoidant → Cluster C → self-esteem → body dissatisfaction → bulimia	.01	.00, .02
Avoidant → Cluster C → self-esteem → body dissatisfaction → drive for thinness	.02	.00, .04
Avoidant → Cluster B → impulse → body dissatisfaction → drive for thinness	.01	.00, .03
Avoidant → cluster C → NSSI	.03	.00, .07
Avoidant → Cluster B → instability → NSSI	.01	.00, .03
Avoidant → Cluster C → instability → NSSI	.01	.00, .02
Avoidant → Cluster B → impulse → NSSI	.02	.00, .04

Only significant mediation paths are shown above. For the full list of mediation paths, please contact the corresponding author

Results showed a significant total indirect effect of avoidant attachment to bulimia (*ß* = 0.09, 99% CIs: 0.02, 0.20), with drive for thinness (*ß* = 0.17, 99% CIs: 0.04, 0.31), and NSSI (*ß* = 0.08, 99% CIs: 0.01, 0.19) showing significant indirect effects. These relationships were the same as for anxious attachment apart that for NSSI self -esteem was a significant mediator.

#### Community group

Inspection of the indirect effects from the attachment variables to ED outcomes showed that the total indirect effects of anxious attachment on bulimia (*ß* = 0.22, 99% CIs: 0.16, 0.29), drive for thinness (*ß* = 0.20, 99% CIs: 0.14, 0.26), and NSSI (*ß* = 0.24, 99% CIs: 0.17, 0.30) were significant in the community group. As shown in Table 4, the relationship between a.) anxious attachment and bulimia and b.) anxious attachment and drive for thinness were mediated by Cluster B and C schemas, self-esteem, impulsivity, and body dissatisfaction. The relationship between anxious attachment and NSSI was mediated by affect dysregulation, self-esteem, and Cluster B and C schemas.

The total indirect effects of avoidant attachment on bulimia symptoms (*ß* = 0.06, 99% CIs: 0.03, 0.10), drive for thinness (*ß* = 0.05, 99% CIs: 0.02, 0.08), and NSSI (*ß* = 0.07, 99% CIs: 0.02, 0.12) were also significant. These relationships were the same as for anxious attachment apart that for NSSI, impulsivity and not self-esteem was a significant mediator.

## Discussion

Informed by the conceptual model of risk proposed by Svirko and Hawton [3] and Claes and Muehlencamp [2], this study examined a novel integrative model of NSSI and ED symptoms in a clinical ED and community group. Our model provided a good fit to the data in the clinical ED sample, but a poor fit in the community sample, suggesting the model does not generalise across groups, or the spectrum of severity for EDs and NSSI. For the community sample only a revised model, which included several additional pathways (e.g., anxious attachment to affect dysregulation and self-esteem, and maladaptive Cluster C schemas directly to NSSI), achieved an acceptable fit. Therefore, the initial proposed model required increased complexity to account for the correlations between ED and NSSI symptoms in a community sample. The significance of predictors differed between the clinical ED group and the community sample, with affect dysregulation being a unique contributor for NSSI in both the ED and community groups. The only shared factor for NSSI and bulimic symptoms in the community, but not the ED sample, was impulsivity.

### Unique and shared predictors for NSSI, bulimia, and drive for thinness

We found that affect dysregulation was a unique predictor for NSSI, but not bulimia or drive for thinness in both the ED and the community groups. Several studies have also revealed positive associations between NSSI and anxiety and depression, often regarded as proxies for affect dysregulation, in ED patients [45, 46]. Regarding community samples, a study using path analysis found that emotion dysregulation predicted positive and negative affect after engaging in NSSI. However, other studies have found positive, not negative affect was responsible for more subsequent lifetime NSSI behaviours [47]. These findings indicate that future studies would benefit from assessing the distinctive roles of both negative and positive affect in predicting NSSI.

Previous models that assessed parts of our proposed integrative model, Muehlenkamp et al. [45] also revealed that NSSI was related to depression through dissociation, whereas disordered eating was motivated by body dissatisfaction. This is also partially in line with Selby and Joiner’s [48] emotional cascade model proposed for borderline personality disorders, which proposes that negative emotions and behavioural dysregulation are interconnected by a self-preserving cycle of rumination, negative thinking patterns, and negative affect. To reduce these aversive emotions, dysregulated behaviours such as NSSI may be used as distractions from extreme rumination. However, our model was limited to support the entire emotional cascade model because rumination was not included as an additional mediating variable.

Contradicting previous studies, which have suggested that disordered eating may also function to regulate emotions [49], in the current study affect dysregulation was not related to any of the ED symptoms. This finding might be because the measurement of affect dysregulation in the current study was more general and not specific to regulating body image dissatisfaction, which may explain why it was not a significant predictor in our path-analyses. Overall, our findings indicate that a more general emotional risk factor (i.e., affect dysregulation) may be related explicitly to NSSI in EDs, but that this factor may not be sufficiently body focused to influence ED behaviours.

Surprisingly, impulsivity was a significant unique predictor of NSSI, and it was also a shared factor for bulimic symptoms and NSSI in the community, but not in the ED group. While this finding partially supports the findings of previous non-clinical ED population studies, which revealed positive correlations between NSSI, impulsiveness, and eating pathology [50], it contradicts other studies that have reported shared contributing factors for impulsivity and NSSI in clinical ED patients, especially in binge/purging ED subtypes [1]. The non-significant finding for impulsivity for the ED group may be attributable to the predominantly restrictive symptomatology (i.e., AN-Restrictive) present in this group [51]. It is also worth noting that apart from impulsivity being shared between NSSI and bulimic symptoms in the community group, no other shared factors between NSSI and ED symtpoms were observed.

In the community group schemas relating to Cluster C personality disorders, were directly associated with NSSI. This finding is consistent with the findings by Anderson et al. [4], which revealed that experiential avoidance, a proxy for Cluster C personality disorders, was the most influential contributing factor to NSSI in their community sample. Insecure attachment may lead individuals to be socially avoidant, and steer away from close involvement with others to protect themselves against anticipated rejection, which then contributes to the adoption of maladaptive coping strategies, such as NSSI, to manage these difficulties [52].

Concerning the ED related outcome variables, our findings indicated a direct relationship between impulsiveness and body dissatisfaction for bulimic symptoms, for the ED and community groups. Furthermore, for the community sample, self-esteem was also a significant predictor for bulimic symptoms. This finding is in accordance with other studies that have shown impulsiveness, low self-esteem, and body dissatisfaction to be important triggering and maintaining factors for bulimic symptoms in both clinical [25] and community [33, 53] samples.

Finally, we observed that low self-esteem and body dissatisfaction were related to drive for thinness in the ED group, which is in line with previous research [54]. However, for the community group, the only significant direct contributor to ED symptoms was body dissatisfaction. Overall body dissatisfaction appears to be the most significant factor for ED symptoms in both the clinical ED and community sample, a finding that has been supported by a previous meta-analyses [32] and more recent longitudinal research [55] on the most proximal risk factors for EDs.

### Variance accounted for in NSSI, bulimia and drive for thinness.

Results showed that our model explained a higher amount of variance for NSSI, bulimia and drive for thinness than previous studies [4, 45]. This was the case for the models assessing the ED and the community samples. The variance explained for NSSI in our path-analyses was 29% for the ED group and 24% for the community group. For the ED outcome variables, the variance ranged from 33% for bulimic symptoms and 57% for drive for thinness for the ED group. For the community group, these values ranged from 39% for bulimic symptoms to 51% for drive for thinness. Our percentages are almost double the amount of variance explained for by NSSI and ED symptoms in other studies [4]. The observed lower variance in bulimic symptoms (*3*3% vs 39%) for the clinical ED sample could be again attributable to the fact that the current ED sample was mainly composed of AN-Restrictive patients (only 38% of the ED sample presented with binge or purging symptoms). Overall, it appears that including insecure attachment and early maladaptive schemas in our model enhanced our understanding of the processes underlying ED symptoms and NSSI.

Despite our model accounting for significantly more variance in our primary outcome variables than other studies, a large amount of variance was still unexplained. Future studies should examine important predisposing factors including aversive childhood experiences, family characteristics (e.g., parenting styles) as well as more immediate emotional (e.g., dissociation) and cognitive (e.g., need for control) variables that have been outlined in the original theoretical models of the co-occurrence of ED symptoms and NSSI [2, 3]. However, increased involvedness in our theoretical understanding of these processes comes with increased statistical complexity, which makes model fitting extremely difficult.

### Indirect effects of insecure attachment and maladaptive schemas

In the current study, the mediation analyses revealed that both anxious and avoidant attachment were related to both NSSI and ED symptoms through maladaptive Cluster C and B schemas as well as the emotional variables (e.g., impulsivity, affect instability, self-esteem). This finding indicates that early in illness progression, there may be a range of general risk factors that relate to both NSSI and ED symptoms. However, as the illness progresses, associations may become narrower and more specific, which can be seen in the fact that apart from impulsivity in the community sample, no other immediate factors were shared between NSSI and ED symptoms. This notion is in line with staging models for mental illnesses such as psychosis [56] and more recently also EDs [57].

The indirect relationships between insecure attachment, maladaptive schemas, and the other emotional variables, are in line with previous studies that have assessed borderline personality disorder patients, where NSSI and ED symptoms commonly co-occur [58]. While both attachment anxiety and attachment avoidance have been implicated in borderline personality disorders [59], there is some research to suggest that the relationship between attachment and borderline personality disorder may be indirect, mediated by factors such as impulsivity, aggression, and trait negative affect [58]. Similarly, in ED patients, a recent review of 22 studies has shown various mediators were responsible for the relationship between attachment and ED symptoms with emotion dysregulation and depression showing the highest effect sizes [60].

These indirect findings might be explained by the fact that NSSI and ED symptoms frequently occur in interpersonal contexts. Caregiver neglect and traumatic events during childhood are influential risk factors for NSSI behaviours in ED populations [12, 15, 23]. These difficult early experiences can lead to the development of insecure attachment styles, which manifest as either avoidant or anxious forms of attachment [61]. Attachment difficulties may predispose an individual to fear negative social evaluation [17], including schemas related to Cluster B (e.g., emotional deprivation) and Cluster C (e.g., failure) personality disorders [11]. In adulthood, these core beliefs remain dormant until they are activated by situations or life events that are relevant to that specific schema [22]. These factors may then become central in triggering both ED and NSSI symptoms through other emotional and body image-related variables, and inevitably serve to maintain both problem sets.

### Limitations

Our findings must be considered within the context of the study's limitations. First, as the data is cross-sectional, future research may benefit from the use of longitudinal designs, which may include constructs such as attachment and schemas earlier in life. It is also important to highlight that our model tested for independent associations for NSSI and ED symptoms. If a variable accounted for some common, shared variance between NSSI and ED symptoms, this was unfortunately not captured by our current model. For instance, it is possible that ED symptoms lead to NSSI, as individuals try to find an alternative coping style to ED behaviours or struggle with associated shame and distress. The idea that NSSI and ED symptoms may be more causally linked in this way, could not be considered in the current study due to the cross-sectional data.

Second, the ED diagnoses were derived from both self and clinician reports, which might have caused some variations in the reliability of the derived ED diagnoses. Furthermore, our recruitment method allowed for the involvement of participants at different stages of their illness/recovery. Our ED sample therefore differed in terms of illness status, with 35% of the sample already recovered from their ED. This recovered sample may have dampened effects in our analyses. Similarly, the clinical ED participants who were in treatment at the time of assessment may have been more likely to engage in impression management when completing the self-report measures.

Third, due to the small sample size of the current versus lifetime ED diagnoses and the ED subtypes we were not able to run separate groups for these groups. In relation to the ED diagnoses, it should be noted that the ED sample predominantly comprised AN-Restrictive, which may have influenced the results of the path analysis for the disordered eating variable of bulimia. This was due to recruitment from tertiary facilities where most of the ED patients had an AN-Restrictive diagnosis and were admitted because of their low weight and associated medical instability. Future research may consider the use of a more balanced currently ill ED sample with equal distribution of participants across all ED subtypes, to determine any existing differences in the variables of interest between ED subtypes.

Fourth, our integrative model for NSSI and ED symptoms was limited to females living in Australia, and we, therefore, do not know whether our findings are generalisable to males and individuals from other countries. Upcoming research would benefit from replicating the current model in clinical and community male populations from different countries.

Fifth, we found significant differences between the clinical ED and control group in terms of ethnicity, age, BMI, and marital and employment status. These differences were most likely because the community sample was primarily ascertained from a university undergraduate course, while the clinical ED population were recruited from inpatient/ outpatient services and organisations from different geographic settings within Australia. Our path models did control for age; however, they did not control for any of the other sociodemographic differences between the ED and community groups. This decision was based to preserve model parsimony. However, it should be noted that the results for our unadjusted and adjusted analyses assessing the differences in all the variables included in our integrative model between the ED and the community sample were the same. This indicates that any differences observed in our models were unlikely due to the demographic differences between the ED and community groups.

Sixth, there are limitations in the conceptualisation of the model such that the current variables may not fully explain the variance in NSSI and ED symptoms indicating other contributing factors. Future research may, therefore, continue to assess variables related to both problem behaviours, including other variables that have recently been implicated in both ED and NSSI, such as rejection sensitivity [19], social rank [19], and alexithymia [62].

Seventh, our impulsivity scale had a low Cronbach alpha value of 0.69 and 0.60 respectively for the ED and the community sample. However, when simulating what results would look like if the measure had better internal consistency, we found that the results of our path-models did not change with improved Cronbach alpha values.

Withstanding these limitations, the proposed conceptual model is the first to assess a wide range of interpersonal, cognitive, and emotional factors known to be associated with both NSSI and ED, in an ED as well as a community sample.

### Clinical implications

The current findings highlight the importance of screening for NSSI within clinical ED populations, and for clinicians to formulate the overlapping and distinct processes which contribute to both problem sets. Knowledge of the shared contributing factors between EDs and NSSI as well as the functional role self-harm and disordered eating may serve for individuals, may contribute to improved clinical decision-making regarding treatment and support. Specifically, those presenting with comorbidity may benefit from treatments that target both ED and NSSI symptoms such as dialectical behavioural therapy [63], schema therapy [22], interpersonal therapy [64], and emotion regulation training programs [65]. Finally, there is the need to address the role of insecure attachment, both through the prevention of insecure attachment and maladaptive schemas using early intervention parenting programs, as well as fostering secure attachment for those already engaged in therapy. The results also highlight the importance of identifying those risk factors more pertinent for individuals with less severe disordered eating such as those participants within the community, to identify where to target prevention strategies.

### Conclusions

In conclusion, the current study expanded upon previous conceptual models of NSSI in EDs. Using an attachment framework, we examined the shared contributions of interpersonal, cognitive, and emotional difficulties which may lead to ED symptoms and NSSI in an ED and a community sample. The results from our path-analyses found that our model provided a good fit for the ED, but not the community sample, indicating that the model was not directly generalisable to a community sample. Our distal factors, of attachment and maladaptive schemas included early in the model were found to be indirectly related to our outcome variables of NSSI and ED symptoms. In terms of our mediating variables, included in the middle of the model, except for impulsivity within the community sample, we did not find other shared vulnerability factors for NSSI and ED symptoms. Future research may consider extending the proposed models to gain further understanding of interpersonal, cognitive, and emotional difficulties associated with EDs and NSSI. Testing these models longitudinally in comparable samples recruited uniformly may help to differentiate the proximal versus distal risk factors included in our proposed model.

## Supplementary Information

**Additional file 1**. Evaluation of the impact of low reliability of impulsivity measure on path analyses.

## Data Availability

The datasets during and/or analysed during the current study available from the corresponding author on reasonable request.

## Abbreviations

NSSI: Non-suicidal self-injury
ED: Eating Disorder
AN: Anorexia Nervosa
DSM-5: Diagnostic and Statistical Manual of Mental Disorders
SD: Standard Deviation
BMI: Body Mass Index
ECR-R: Revised Experiences in Close Relationships scale
YSQ-SF: Young Schema Questionnaire – Short Form
BPQ: Borderline Personality Questionnaire
EDI-3: Eating Disorder Inventory 3
CFI: Comparative Fit Index
RMSEA: Root Mean Square Error of Approximation
SRMR: Standardised Root Mean Residual

## References

[1] Cucchi A, Ryan D, Konstantakopoulos G, Stroumpa S, Kacar AS, Renshaw S (2016). Lifetime prevalence of non-suicidal self-injury in patients with eating disorders: a systematic review and meta-analysis. Psychol Med.

[2] Claes L, Muehlenkamp JJ (2014). Non-suicidal self-injury in eating disorders.

[3] Svirko E, Hawton K (2007). Self-injurious behavior and eating disorders: the extent and nature of the association. Suicide Life Threat Behav.

[4] Anderson NL, Smith KE, Mason TB, Crowther JH (2018). Testing an integrative model of affect regulation and avoidance in non-suicidal self-injury and disordered eating. Arch Suicide Res.

[5] Muehlenkamp JJ, Claes L, Smits D, Peat CM, Vandereycken W (2011). Non-suicidal self-injury in eating disordered patients: a test of a conceptual model. Psychiatry Res.

[6] Vieira A, Machado B, Moreira C, Machado P, Brandão I, Roma-Torres A (2018). Eating disorders and non-suicidal self-injury: structural equation modelling of a conceptual model. Eur Eat Disord Rev.

[7] Glazebrook K, Townsend E, Sayal K (2015). The role of attachment style in predicting repetition of adolescent self-harm: a longitudinal study. Suicide Life-Threat Behav.

[8] Waller G (2003). Schema-level cognitions in patients with binge eating disorder: a case control study. Int J Eat Disord.

[9] Claes L, Vandereycken W, Vertommen H (2001). Self-injurious behaviors in eating-disordered patients. Eat Behav.

[10] Solano R, Fernández-Aranda F, Aitken A, López C, Vallejo J (2005). Self-injurious behaviour in people with eating disorders. Eur Eat Disord Rev.

[11] Pauwels E, Dierckx E, Schoevaerts K, Claes L (2016). Early maladaptive schemas in eating disordered patients with or without non-suicidal self-injury. Eur Eat Disord Rev.

[12] Vieira A, Ramalho S, Brandao I, Saraiva J, Goncalves S (2016). Adversity, emotion regulation, and non-suicidal self-injury in eating disorders. Eat Disord.

[13] Iannaccone M, Cella S, Manzi SA, Visconti L, Manzi F, Cotrufo P (2013). My body and me: self-injurious behaviors and body modifications in eating disorders–preliminary results. Eat Disord.

[14] Turner BJ, Yiu A, Layden BK, Claes L, Zaitsoff S, Chapman AL (2015). Temporal associations between disordered eating and nonsuicidal self-injury: examining symptom overlap over 1 year. Behav Ther.

[15] Kostro K, Lerman JB, Attia E (2014). The current status of suicide and self-injury in eating disorders: a narrative review. J Eat Disord.

[16] MacLaren VV, Best LA (2010). Nonsuicidal self-injury, potentially addictive behaviors, and the five factor model in undergraduates. Pers Individ Differ.

[17] Arcelus J, Haslam M, Farrow C, Meyer C (2013). The role of interpersonal functioning in the maintenance of eating psychopathology: a systematic review and testable model. Clin Psychol Rev.

[18] Caglar-Nazali HP, Corfield F, Cardi V, Ambwani S, Leppanen J, Olabintan O (2014). A systematic review and meta-analysis of ‘Systems for Social Processes’ in eating disorders. Neurosci Biobehav Rev.

[19] De Paoli T, Fuller-Tyszkiewicz M, Krug I (2017). Insecure attachment and maladaptive schema in disordered eating: the mediating role of rejection sensitivity. Clin Psychol Psychother.

[20] Kimball JS, Diddams M (2007). Affect regulation as a mediator of attachment and deliberate self-harm. J Coll Couns.

[21] Levesque C, Lafontaine M-F, Bureau J-F, Cloutier P, Dandurand C (2010). The influence of romantic attachment and intimate partner violence on non-suicidal self-injury in young adults. J Youth Adolesc.

[22] Young JE, Klosko JS, Weishaar ME (2003). Schema therapy: a practitioner’s guide.

[23] Voderholzer U, Schwartz C, Thiel N, Kuelz AK, Hartmann A, Scheidt CE (2014). A comparison of schemas, schema modes and childhood traumas in obsessive-compulsive disorder, chronic pain disorder and eating disorders. Psychopathology.

[24] Young JE. Cognitive therapy for personality disorders: a schema-focused approach. 3 ed. Sarasota: Professional Resource Exchange; 1999.

[25] Dingemans A, Spinhoven P, Van Furth E (2006). Maladaptive core beliefs and eating disorder symptoms. Eat Disord.

[26] Leung N, Waller G, Thomas G (1999). Core beliefs in anorexic and bulimic women. J Nerv Ment Dis.

[27] APA. American Psychiatric Association. Diagnostic and statistical manual of mental disorders (DSM-5®). American Psychiatric Pub., 2013.

[28] Todisco P, Meneguzzo P, Garolla A, Antoniades A, Vogazianos P, Tozzi F (2021). Impulsive behaviors, and clinical outcomes following a flexible intensive inpatient treatment for eating disorders: findings from an observational study. Eat Weight Disord.

[29] Unoka Z, Tölgyes T, Czobor P, Simon L (2010). Eating disorder behavior and early maladaptive schemas in subgroups of eating disorders. J Nerv Ment Dis.

[30] Arthurs SD, Tan JC (2017). Personality traits, early maladaptive schemas, and severity of nonsuicidal self-injury. Psi Chi J Psychol Res.

[31] Peck LD, Lightsey OR (2008). The eating disorders continuum, self-esteem, and perfectionism. J Couns Dev.

[32] Stice E (2002). Risk and maintenance factors for eating pathology: a meta-analytic review. Psychol Bull.

[33] Stice E, Desjardins CD (2018). Interactions between risk factors in the prediction of onset of eating disorders: Exploratory hypothesis generating analyses. Behav Res Ther.

[34] Striegel-Moore RH, Rosselli F, Perrin N, DeBar L, Wilson GT, May A (2009). Gender difference in the prevalence of eating disorder symptoms. Int J Eat Disord.

[35] Fraley RC, Waller NG, Brennan KA (2000). An item response theory analysis of self-report measures of adult attachment. J Pers Soc Psychol.

[36] Sibley CG, Fischer R, Liu JH (2005). Reliability and validity of the revised experiences in close relationships (ECR-R) self-report measure of adult romantic attachment. Pers Soc Psychol Bull.

[37] Poreh AM, Rawlings D, Claridge G, Freeman JL, Faulkner C, Shelton C (2006). The BPQ: a scale for the assessment of borderline personality based on DSM-IV criteria. J Pers Disord.

[38] Fonseca-Pedrero E, Paino M, Lemos-Giraldez S, Sierra-Baigrie S, Gonzalez MP, Bobes J (2011). Borderline personality traits in nonclinical young adults. J Pers Disord.

[39] Garner DM. Eating disorder inventory-3 (EDI-3). Professional manual. Odessa, FL: Psychological Assessment Resources; 2004.

[40] Clausen L, Rosenvinge JH, Friborg O, Rokkedal K (2011). Validating the eating disorder inventory-3 (EDI-3): a comparison between 561 female eating disorders patients and 878 females from the general population. J Psychopathol Behav Assess.

[41] Cohen J (1988). Statistical power analysis for the behavioral sciences.

[42] Schermelleh-Engel K, Moosbrugger H, Müller H (2003). Evaluating the fit of structural equation models: tests of significance and descriptive goodness-of-fit measures. Psychol Methods.

[43] Shrout PE, Bolger N (2002). Mediation in experimental and nonexperimental studies: new procedures and recommendations. Psychol Methods.

[44] MacCallum RC, Widaman KF, Zhang S, Hong S (1999). Sample size in factor analysis. Psychol Methods.

[45] Muehlenkamp JJ, Peat CM, Claes L, Smits D (2012). Self-injury and disordered eating: expressing emotion dysregulation through the body. Suicide Life Threat Behav.

[46] Greene D, Boyes M, Hasking P (2020). The associations between alexithymia and both non-suicidal self-injury and risky drinking: a systematic review and meta-analysis. J Affect Disord.

[47] Jenkins AL, Schmitz MF (2012). The roles of affect dysregulation and positive affect in non-suicidal self-injury. Arch Suicide Res.

[48] Selby EA, Anestis MD, Bender TW, Joiner TE (2009). An exploration of the emotional cascade model in borderline personality disorder. J Abnorm Psychol.

[49] Ambwani S, Roche MJ, Minnick AM, Pincus AL (2015). Negative affect, interpersonal perception, and binge eating behavior: an experience sampling study. Int J Eat Disord.

[50] Black EB, Mildred H (2014). A cross-sectional examination of non-suicidal self-injury, disordered eating, impulsivity, and compulsivity in a sample of adult women. Eat Disord.

[51] Wagner AF, Vitousek KM (2019). Personality variables and eating pathology. Psychiatr Clin North Am.

[52] Bartholomew K, Horowitz LM (1991). Attachment styles among young adults: A test of a four-category model. J Pers Soc Psychol.

[53] Hovrud L, Simons R, Simons J, Korkow J (2020). Non-suicidal self-injury and bulimia: the role of emotion dysregulation and body dissatisfaction. Eat Weight Disord.

[54] Puttevils L, Vanderhasselt MA, Vervaet M (2019). Investigating transdiagnostic factors in eating disorders: does self-esteem moderate the relationship between perfectionism and eating disorder symptoms?. Eur Eat Disord Rev.

[55] Stice E, Van Ryzin MJ (2019). A prospective test of the temporal sequencing of risk factor emergence in the dual pathway model of eating disorders. J Abnorm Psych.

[56] McGorry PD, Nelson B, Goldstone S, Yung AR (2010). Clinical staging: a heuristic and practical strategy for new research and better health and social outcomes for psychotic and related mood disorders. Can J Psychiatry.

[57] Treasure J, Cardi V, Leppanen J, Turton R (2015). New treatment approaches for severe and enduring eating disorders. Physiol Behav.

[58] Scott LN, Levy KN, Pincus AL (2009). Adult attachment, personality traits, and borderline personality disorder features in young adults. J Pers Disord.

[59] Levy KN (2005). The implications of attachment theory and research for understanding borderline personality disorder. Dev Psychopathol.

[60] Cortés-García L, Takkouche B, Seoane G, Senra C (2019). Mediators linking insecure attachment to eating symptoms: a systematic review and meta-analysis. PLoS ONE.

[61] Bowlby J. Attachment and loss. Separation: anxiety and anger. II. New York: Basic Books; 1973.

[62] Giles S, Hughes EK, Fuller-Tyszkiewicz M, Krug I (2020). The cognitive-interpersonal model of disordered eating: a test of the mediating role of alexithymia. Eur Eat Disord Rev.

[63] Linehan MM (1987). Dialectical behavior therapy for borderline personality disorder: theory and method. Bull Menninger Clin.

[64] Rieger E, Van Buren DJ, Bishop M, Tanofsky-Kraff M, Welch R, Wilfley DE (2010). An eating disorder-specific model of interpersonal psychotherapy (IPT-ED): causal pathways and treatment implications. Clin Psychol Rev.

[65] Holmqvist Larsson K, Andersson G, Stern H, Zetterqvist M (2020). Emotion regulation group skills training for adolescents and parents: A pilot study of an add-on treatment in a clinical setting. Clin Child Psychol Psychiatry.

