# Supplementary material for: A new integrative model for the co-occurrence of non-suicidal self-injury behaviours and eating disorder symptoms
Source: J Eat Disord. 2021 Nov 22;9:153. doi: 10.1186/s40337-021-00508-3 (PMC8607624; doi:10.1186/s40337-021-00508-3)
Supplement: Supplementary file 1 — Additional file 1. Evaluation of the impact of low reliability of impulsivity measure on path analyses. [file 40337_2021_508_MOESM1_ESM.docx]

**Supplementary analyses 1: Evaluation of the impact of low reliability of impulsivity measure on path analyses**

Correlations involving impulsivity were disattenuated by evaluating what the correlation size would be if reliability for impulsivity was 0.80 or 0.90 instead of current values (0.69 for ED sample and 0.60 for community sample). As shown below, the results for path analyses are very similar, even with improved reliability.

**ED sample (*n* = 123)**

Table S.1 – *overall model fit as a function of disattenuated correlations involving impulsivity*

|  | X^2^ | df | p | CFI | RMSEA | SRMR |
| --- | --- | --- | --- | --- | --- | --- |
| Original model |  |  |  |  |  |  |
| Alpha = 0.69 | 25.22 | 26 | .507 | 1.000 | .000 | .032 |
| Alpha = 0.80 | 30.62 | 26 | .243 | .993 | .038 | .036 |
| Alpha = 0.90 | 30.72 | 26 | .239 | .993 | .038 | .037 |

Table S.2 – *variance explained in outcome variables as a function of disattenuated correlations involving impulsivity*

|  | R-squared | | |
| --- | --- | --- | --- |
| Variable | Alpha = 0.69 | Alpha = 0.80 | Alpha = 0.90 |
| Body dissatisfaction | .520 | .532 | .526 |
| Bulimia | .331 | .351 | .334 |
| Drive for thinness | .569 | .576 | .576 |
| Self-mutilation | .291 | .288 | .286 |
| Impulsivity | .196 | .246 | .217 |
| Self esteem | .558 | .559 | .559 |
| Instability | .466 | .470 | .470 |
| Cluster B | .389 | .393 | .393 |
| Cluster C | .455 | .449 | .449 |

Table S.3 - *Standardised coefficients from path analyses*

|  | Alpha = 0.69 | | Alpha = 0.80 | | Alpha = 0.90 | |
| --- | --- | --- | --- | --- | --- | --- |
| Pathway | *ß* | p | *ß* | p | *ß* | p |
| Self-mutilation on Instability | 0.312 | .003 | 0.309 | .001 | 0.312 | .001 |
| Self-esteem | -0.108 | .372 | -0.101 | .308 | -0.104 | .294 |
| Impulsivity | 0.149 | .059 | 0.151 | .074 | 0.138 | .101 |
| Body dissatisfaction | 0.142 | .249 | 0.132 | .233 | 0.140 | .203 |
| Age | -0.237 | .003 | -0.235 | .003 | -0.237 | .003 |
|  |  |  |  |  |  |  |
| Bulimia on Instability | -0.017 | .849 | -0.023 | .803 | -0.015 | .870 |
| Self-esteem | -0.033 | .686 | -0.023 | .813 | -0.030 | .759 |
| Impulsivity | 0.328 | <.001 | 0.363 | <.001 | 0.330 | <.001 |
| Body dissatisfaction | 0.329 | <.001 | 0.301 | .004 | 0.322 | .002 |
| Age | -0.168 | .009 | -0.162 | .034 | -0.167 | .031 |
|  |  |  |  |  |  |  |
| Drive for thinness on Self-esteem | 0.295 | .001 | 0.287 | <.001 | 0.287 | <.001 |
| Body dissatisfaction | 0.531 | <.001 | 0.541 | <.001 | 0.541 | <.001 |
| Age | -0.027 | .641 | -0.034 | .570 | -0.034 | .570 |
|  |  |  |  |  |  |  |
| Body dissatisfaction on Impulsivity | 0.234 | <.001 | 0.268 | <.001 | 0.254 | <.001 |
| Instability | 0.306 | <.001 | 0.287 | <.001 | 0.296 | <.001 |
| Self-esteem | 0.434 | <.001 | 0.431 | <.001 | 0.432 | <.001 |
| Age | 0.078 | .219 | 0.088 | .176 | 0.086 | .188 |
|  |  |  |  |  |  |  |
| Impulsivity on Cluster B | 0.432 | <.001 | 0.479 | <.001 | 0.449 | <.001 |
| Cluster C | -0.005 | .963 | 0.001 | .993 | 0.003 | .981 |
| Age | -0.067 | .336 | -0.078 | .328 | -0.071 | .379 |
|  |  |  |  |  |  |  |
| Self-esteem on Cluster B | -0.016 | .825 | -0.009 | .914 | -0.009 | .914 |
| Cluster C | 0.733 | <.001 | 0.728 | <.001 | 0.728 | <.001 |
| Age | -0.103 | .089 | -0.107 | .077 | -0.107 | .077 |
|  |  |  |  |  |  |  |
| Instability on Cluster B | 0.325 | <.001 | 0.339 | <.001 | 0.339 | <.001 |
| Cluster C | 0.360 | <.001 | 0.347 | <.001 | 0.347 | <.001 |
| Age | -0.182 | .003 | -0.187 | .005 | -0.187 | .005 |
|  |  |  |  |  |  |  |
| Cluster B on Anxious | 0.437 | <.001 | 0.441 | <.001 | 0.441 | <.001 |
| Avoidant | 0.279 | .004 | 0.278 | .001 | 0.278 | .001 |
| Age | 0.016 | .812 | 0.017 | .817 | 0.017 | .817 |
|  |  |  |  |  |  |  |
| Cluster C on Anxious | 0.439 | <.001 | 0.444 | <.001 | 0.444 | <.001 |
| Avoidant | 0.322 | .001 | 0.321 | <.001 | 0.321 | <.001 |
| Age | -0.014 | .842 | -0.014 | .840 | -0.014 | .840 |
|  |  |  |  |  |  |  |
| Self-mutilation with Bulimia | 0.009 | .912 | -0.009 | .923 | 0.002 | .982 |
| Drive for thinness | 0.175 | .051 | 0.141 | .110 | 0.145 | .101 |
|  |  |  |  |  |  |  |
| Bulimia with Drive for thinness | 0.196 | .019 | 0.112 | .210 | 0.120 | .178 |
|  |  |  |  |  |  |  |
| Cluster B with cluster C | 0.462 | <.001 | 0.451 | <.001 | 0.451 | <.001 |
|  |  |  |  |  |  |  |
| Impulsivity with instability | 0.009 | .926 | -0.001 | .991 | -0.003 | .978 |

**Community sample (*n* = 531)**

Table S.4 – *overall model fit as a function of disattenuated correlations involving impulsivity*

|  | X2 | df | P | CFI | RMSEA | SRMR |
| --- | --- | --- | --- | --- | --- | --- |
| Original model |  |  |  |  |  |  |
| Alpha = 0.69 | 144.977 | 26 | <.001 | .949 | .093 | .054 |
| Alpha = 0.80 | 157.009 | 26 | <.001 | .950 | .097 | .060 |
| Alpha = 0.90 | 158.071 | 26 | <.001 | .949 | .098 | .061 |
| Revised model |  |  |  |  |  |  |
| Alpha = 0.69 | 51.818 | 22 | <.001 | .987 | .051 | .033 |
| Alpha = 0.80 | 55.276 | 22 | <.001 | .987 | .053 | .037 |
| Alpha = 0.90 | 55.169 | 22 | <.001 | .987 | .053 | .037 |

Table S.5 – *variance explained in outcome variables in revised model as a function of disattenuated correlations involving impulsivity*

|  | R-squared | | |
| --- | --- | --- | --- |
| Variable | Alpha = 0.69 | Alpha = 0.80 | Alpha = 0.90 |
| Body dissatisfaction | .274 | .282 | .279 |
| Bulimia | .388 | .402 | .392 |
| Drive for thinness | .505 | .504 | .504 |
| NSSI | .236 | .242 | .242 |
| Impulsivity | .163 | .199 | .180 |
| Self esteem | .530 | .535 | .535 |
| Instability | .364 | .363 | .363 |
| Cluster B | .314 | .310 | .310 |
| Cluster C | .288 | .295 | .295 |

Table S.6 - *Standardised coefficients from path analyses*

|  | Alpha = 0.69 | | Alpha = 0.80 | | Alpha = 0.90 | |
| --- | --- | --- | --- | --- | --- | --- |
| Pathway | *ß* | p | *ß* | p | *ß* | p |
| Self-mutilation on Instability | 0.180 | .001 | 0.183 | <.001 | 0.186 | <.001 |
| Self-esteem | 0.106 | .086 | 0.098 | .093 | 0.098 | .091 |
| Impulsivity | 0.150 | .001 | 0.169 | <.001 | 0.165 | <.001 |
| Body dissatisfaction | 0.013 | .793 | 0.009 | .840 | 0.012 | .793 |
| Age | -0.022 | .455 | -0.021 | .581 | -0.019 | .632 |
| Cluster C | 0.186 | .002 | 0.180 | .001 | 0.181 | .001 |
|  |  |  |  |  |  |  |
| Bulimia on Instability | 0.031 | .393 | 0.014 | .718 | 0.027 | .500 |
| Self-esteem | 0.132 | .001 | 0.123 | .004 | 0.125 | .004 |
| Impulsivity | 0.228 | <.001 | 0.270 | <.001 | 0.239 | <.001 |
| Body dissatisfaction | 0.414 | <.001 | 0.403 | <.001 | 0.413 | <.001 |
| Age | -0.053 | .053 | -0.060 | .080 | -0.053 | .125 |
|  |  |  |  |  |  |  |
| Drive for thinness on Self-esteem | 0.044 | .233 | 0.040 | .260 | 0.040 | .260 |
| Body dissatisfaction | 0.687 | <.001 | 0.688 | <.001 | 0.689 | <.001 |
| Age | -0.008 | .789 | -0.015 | .619 | -0.015 | .619 |
|  |  |  |  |  |  |  |
| Body dissatisfaction on Impulsivity | 0.179 | <.001 | 0.209 | <.001 | 0.193 | <.001 |
| Instability | 0.070 | .137 | 0.053 | .251 | 0.061 | .186 |
| Self-esteem | 0.405 | <.001 | 0.401 | <.001 | 0.405 | <.001 |
| Age | -0.012 | .770 | -0.014 | .704 | -0.010 | .798 |
|  |  |  |  |  |  |  |
| Impulsivity on Cluster B | 0.429 | <.001 | 0.461 | <.001 | 0.451 | <.001 |
| Cluster C | -0.038 | .583 | -0.024 | .713 | -0.038 | .557 |
| Age | 0.102 | .030 | 0.117 | .003 | 0.103 | .010 |
|  |  |  |  |  |  |  |
| Self-esteem on Cluster B | 0.092 | .069 | 0.092 | .062 | 0.092 | .062 |
| Cluster C | 0.508 | <.001 | 0.508 | <.001 | 0.509 | <.001 |
| Age | -0.035 | .200 | -0.036 | .228 | -0.036 | .228 |
| Anxious | 0.219 | <.001 | 0.220 | <.001 | 0.220 | <.001 |
|  |  |  |  |  |  |  |
| Instability on Cluster B | 0.260 | <.001 | 0.276 | <.001 | 0.274 | <.001 |
| Cluster C | 0.236 | <.001 | 0.217 | <.001 | 0.216 | <.001 |
| Age | -0.027 | .398 | -0.031 | .378 | -0.031 | .378 |
| Anxious | 0.198 | <.001 | 0.198 | <.001 | 0.201 | <.001 |
|  |  |  |  |  |  |  |
| Cluster B on Anxious | 0.425 | <.001 | 0.422 | <.001 | 0.422 | <.001 |
| Avoidant | 0.235 | <.001 | 0.233 | <.001 | 0.233 | <.001 |
| Age | -0.037 | .217 | -0.037 | .303 | -0.037 | .303 |
|  |  |  |  |  |  |  |
| Cluster C on Anxious | 0.443 | <.001 | 0.446 | <.001 | 0.446 | <.001 |
| Avoidant | 0.140 | .001 | 0.144 | <.001 | 0.144 | <.001 |
| Age | -0.114 | <.001 | -0.117 | .002 | -0.117 | .001 |
|  |  |  |  |  |  |  |
| Self-mutilation with Bulimia | 0.019 | .710 | 0.011 | .806 | 0.016 | .717 |
| Drive for thinness | 0.069 | .133 | 0.062 | .149 | 0.61 | .157 |
|  |  |  |  |  |  |  |
| Bulimia with Drive for thinness | 0.353 | <.001 | 0.354 | <.001 | 0.351 | <.001 |
|  |  |  |  |  |  |  |
| Cluster B with cluster C | 0.704 | <.001 | 0.707 | <.001 | 0.707 | <.001 |
|  |  |  |  |  |  |  |
| Impulsivity with instability | 0.186 | <.001 | 0.207 | <.001 | 0.196 | <.001 |
|  |  |  |  |  |  |  |
| Instability with self-esteem | 0.224 | <.001 | 0.230 | <.001 | 0.232 | <.001 |
